# Psmir: a database of potential associations between small molecules and miRNAs

**DOI:** 10.1038/srep19264

**Published:** 2016-01-13

**Authors:** Fanlin Meng, Jing Wang, Enyu Dai, Feng Yang, Xiaowen Chen, Shuyuan Wang, Xuexin Yu, Dianming Liu, Wei Jiang

**Affiliations:** 1 College of Bioinformatics Science and Technology, Harbin Medical University, Harbin 150081, P. R. China

## Abstract

miRNAs are key post-transcriptional regulators of many essential biological processes, and their dysregulation has been validated in almost all human cancers. Restoring aberrantly expressed miRNAs might be a novel therapeutics. Recently, many studies have demonstrated that small molecular compounds can affect miRNA expression. Thus, prediction of associations between small molecules and miRNAs is important for investigation of miRNA-targeted drugs. Here, we analyzed 39 miRNA-perturbed gene expression profiles, and then calculated the similarity of transcription responses between miRNA perturbation and drug treatment to predict drug-miRNA associations. At the significance level of 0.05, we obtained 6501 candidate associations between 1295 small molecules and 25 miRNAs, which included 624 FDA approved drugs. Finally, we constructed the Psmir database to store all potential associations and the related materials. In a word, Psmir served as a valuable resource for dissecting the biological significance in small molecules’ effects on miRNA expression, which will facilitate developing novel potential therapeutic targets or treatments for human cancers. Psmir is supported by all major browsers, and is freely available at http://www.bio-bigdata.com/Psmir/.

Dysregulation of miRNAs has been implicated in a plethora of diseases, giving miRNAs great potential in both cancer diagnostics and treatment. Modulation of miRNA expression levels has been demonstrated as a viable strategy for tumor therapeutics^1^. The well studied miRNA modulators include antisense oligonucleotides (anti-miRs), antagomirs, miRNA sponges, and so on^2^. However, the inefficient delivery into target tissues and suboptimal pharmacodynamics or pharmacokinetics properties are major hurdles in the oligonucleotide-based therapeutics, which highlighting the need and importance for small-molecule-based intervention strategies^3^. Small molecules have indeed immense therapeutic potential to modulate miRNA expression, because they are inclined to possess ideal drug properties, including good solubility, bioavailability and metabolism^4^. Gumireddy *et al.* applied cell-based assays to report small molecule diazobenzene as modifiers for miR-21, suggesting that miR-21 may become a druggable target^5^. Melo *et al.* provided evidence that the small molecule enoxacin modulated miRNA processing by enhancing TRBP (TAR RNA binding protein) and affected miRNA expression^6^.

Many types of public database are available for providing valuable information about miRNAs. HMDD and miR2Disease have shown their great help in functional analysis of miRNAs in various diseases^7^,^8^. miREnvironment mainly fills the gap among miRNA, environmental factors (such as exercise, alcohol, radiation and nutrition) and phenotypes (such as cytotoxicity, fat loss and diseases)^9^. SM2miR is the first manually curated database to comprehensively collect the verified miRNAs response to small molecules^10^. Here, we attempt to develop Psmir database to provide the potential small molecule-miRNA interactions which are inferred through computational algorithm in a large-scale based on similarity of genome-wide expression profiles. Among the predictions, the negative associations indicate that the small molecules may function by inhibiting corresponding miRNAs.

In our previous studies, we have proposed computational approaches to identify the potential connections between small molecules and miRNAs based on gene expression similarity in Alzheimer’s diseases, as well as cancers^11^,^12^. Since gene expression profiles following miRNA perturbation were rare in the past, we simulated the presence and absence in miRNA perturbation using up-regulated and down-regulated miRNA in disease condition. Moreover, we built up-regulated/down-regulated miRNA-specific signature by intersecting differentially expressed genes and target genes. Along with abundance of miRNA transfection experiments, genome-wide data regarding the effects of miRNA on gene expression are available, which facilitates obtaining miRNA-perturbed gene expression profiles. It’s noted that the miRNA transfection experiments refer to promoting or inhibiting one miRNA expression using specific miRNA mimics or inhibitors. To build Psmir, we made efforts to comprehensively collect 39 miRNA affected gene expression profiles from Gene Expression Omnibus (GEO). We incorporated the intrinsic miRNA-specific signatures that were extracted from these miRNA-perturbed gene expression profiles into our computational pipeline. The gene expression profiles measured from the cell lines transfection of miRNA mimics or inhibitors will reflect the miRNA regulation effect more directly, which to some extent promotes the reliability of our prediction.

## Results

We searched the corresponding datasets with the keywords “miRNA transfection” or “microRNA transfection” in GEO database. As a result, 124 miRNA-perturbed gene expression profiles comprising both miRNA-transfected group and control group were manually collected as of Sep. 20th in 2013. At the same time, we obtained 6100 small molecule-transfected gene expression profiles from the Connectivity Map (cmap, build 02, https://www.broadinstitute.org/cmap/)^13^. Here, we only chose the datasets measured by the same platform (HG-U133A) with cmap for further analysis. Finally, we collected 39 miRNA-perturbed and 6100 small molecule-perturbed gene expression profiles involving 25 miRNAs and 1309 small molecules to perform expression profile similarity analysis (details in Methods). All candidate 51051 associations between 1309 small molecules and 39 miRNAs across 25 miRNAs transfecting in different experimental conditions were evaluated. At the significant level of 0.05 and 0.01, the number of significant associations was 6501 and 1937, respectively. The details of associations were provided in our online database known as Psmir.

**Figure 1:**
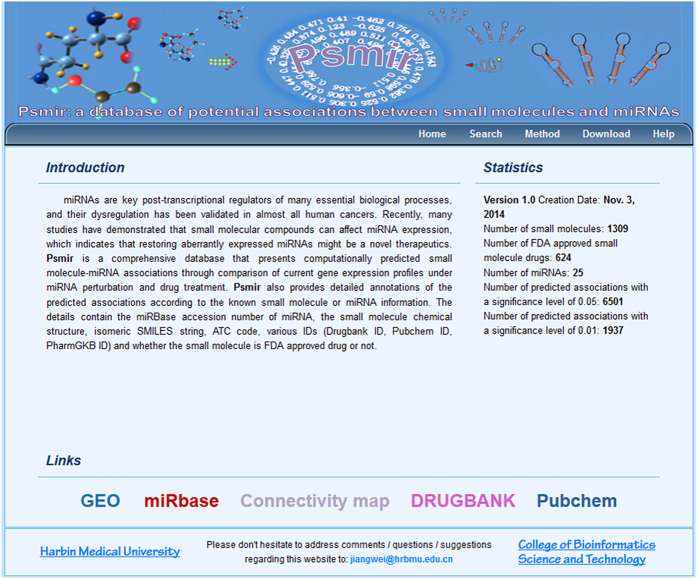
The snapshot of the interface of Psmir database.

Psmir is an accessible web interface for users to browse, search and download small molecule and miRNA associations via a user-friendly graphical user interface at the web address http://www.bio-bigdata.com/Psmir/. A snapshot of the database interface is provided in Fig. 1. In the Search section, by selecting miRNA or small molecule in corresponding dropdown menu, users will retrieve the entries that comprise of small molecule name, miRNA name, condition of the miRNA transfection experiment, *AS* (Association Score) and significance *p*-value. For small molecules, we provide an option that facilitates users focusing on FDA-approved drugs. We further annotated the predicted associations in details according to the known small molecule or miRNA information. The details contain the miRBase accession number of miRNA, the small molecule chemical structure, isomeric SMILES string, ATC code, various IDs (Drugbank ID, Pubchem ID, PharmGKB ID) and whether the small molecule is FDA approved drug or not. The significance of the small molecule and miRNA association was obtained from permutation test.

Psmir is developed in JSP. The Psmir website is deployed in tomcat 6.0.33 web server and runs under Cent OS 5.5 system, supported by a MySQL database to manage data.

## Case Study

**Figure 2:**
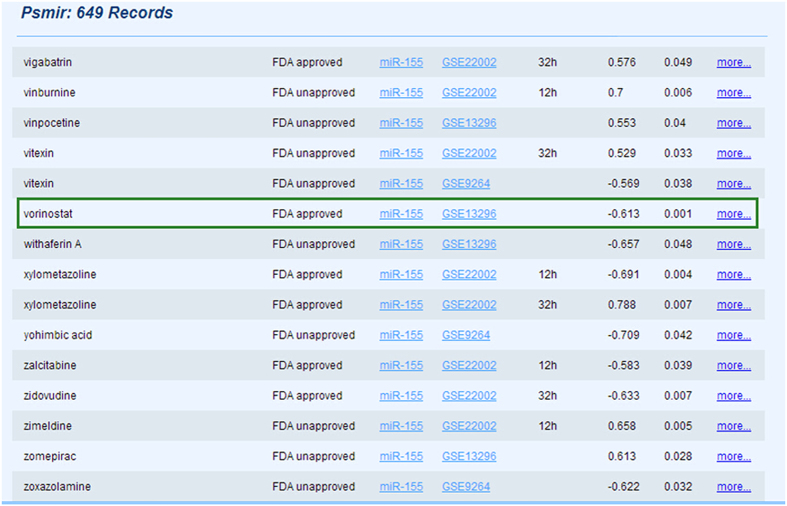
The query results of miR-155. At the significant level of 0.05, we retrieved the 649 records corresponding to miR-155. A record included small molecule name, whether the small molecule is FDA approved drugs or not, miRNA name, the GEOseries ID of the miRNA perturbed expression profiles, the condition of the miRNA transfection experiment, the scores of the associations, the significant *p*-values of the associations and the detail information for the associations.

Taking miR-155 as an example, we selected miR-155 in pull down menu, the result corresponding to miR-155 was illustrated in (Fig. 2). MiR-155 is an oncogenic miRNA which regulates several cancer-related pathways and is most significantly up-regulated miRNA in several cancers, such as breast cancer^14^, colon cancer^15^ and lung cancer^16^. According to the results of Psmir database, we found that the association of miR-155 and vorinostat presented the negative correlation pattern, which meant that these two molecules regulated the opposite pattern on gene expression at the whole genome level. Vorinostat is a histone deacetylase inhibitor which is a drug under investigation for treatment of cutaneous T-cell lymphoma, and also many research reported its anticancer activity over variety of cancers^17^,^18^. In lung cancer, miR-155 is frequently up-regulated^19^, as well as able to predict recurrence and survival^16^. Lee *et al.* indicated that the expression level of miR-155 is obviously decreased following vorinostat treatment^20^. What’s more, the target genes of miR-155 related to angiogenesis, such as AGTR1 and MYD88^21^,^22^, and cell proliferation and differentiation, such as BCL2, FGF7^23^,^24^. These biological processes are closely related to lung cancer. Vorinostat may regulate these target genes of the miRNA and reverse the expression of the miRNA from up-regulation to normal level in lung cancer. Furthermore, the association of miR-155 and vorinostat was also verified in the SM2miR database which is a manually curated database on small molecules’ effects on miRNA expression^10^. Predicting associations between small molecules and miRNAs has potential to provide a valuable biological insight for cancer therapies.

## Discussion

The major goal of the present work was to provide potential drug-miRNA associations, which may be a valuable resource for drug-target studies. In this study, we utilized a computational method to infer small molecule-miRNA associations. Firstly, we collected the gene expression profiles perturbed by miRNAs by keywords “miRNA transfection” or “microRNA transfection” from the GEO database. Meanwhile, we obtained the small molecule-perturbed gene expression profiles from cmap. After screening the probes perturbed by one small molecule or one miRNA, we calculated the degree of the probes perturbed by one small molecule (or one miRNA) significantly distributing at the top/bottom of the entire miRNA-perturbed (or small molecule perturbed) gene expression profile. The size of the score determined the degree of similarity between the small molecule and the miRNA. Next, considering multiple gene expression profiles perturbed by the same small molecule, we combined the scores between the same small molecule and the miRNA. What’s more, we developed a database known as Psmir, which presented all the results. Researchers who are interested in dissecting the potential relationship of pair-wise small molecules and miRNAs could obtain inference from Psmir which comprises statistically significant associations based on high throughput and public data. In order to expand the application, we annotated each entry according to several of the most popular databases such as Drugbank, miRBase and so on. The users can retrieve all records by selecting one miRNA or small molecule in pull down menu. Besides that, users also can obtain more detailed information about miRNA (such as miRBase accession number of miRNA) and small molecule (such as ATC code of drug) in ‘*Detail*’ item.

As far as we know, this is the first database to predict potential associations between small molecules and miRNAs. However, most of human miRNAs have not been designed for transfection experiments, our predicted small molecule-miRNA associations are limited by the number of the available miRNA-perturbed gene expression profiles. With the increasing of miRNA-perturbed or small molecule-perturbed gene expression profiles, we will constantly update Psmir through implementing prediction pipeline for more miRNAs or small molecules in the future.

We believe that Psmir is a valuable complement to the existing curated resources about small molecules and miRNAs, providing a reference to investigations on small molecule and miRNA associations. These associations will facilitate developing novel potential therapeutic targets for human diseases, and may be used to identify drug candidates and predict new indications for existing drugs.

## Methods

### MiRNA-perturbed gene expression profiles

We searched the GEO database by keywords “miRNA transfection” or “microRNA transfection” and obtained the corresponding GEO series (GSEs) IDs and summary of the experiments. We only reserved the GSEs that comprised both miRNA-transfected group and corresponding control group. Finally, 124 miRNA-perturbed gene expression profiles were manually collected as of Sep. 20th in 2013. Individual gene expression profile is measured from specific miRNA mimics/inhibitors transfection into cultured cell lines. In line with the small molecule-perturbed gene expression profiles obtained from the cmap database, we applied 39 miRNA-perturbed gene expression profiles corresponding to Affymetrix HG-U133-generation microarrays, involving 25 different miRNAs and more than 10 cancer cell lines (Table S1). For simplicity, different cell lines, time points and doses for the same miRNA in different experiments are considered as independent gene expression profiles in the subsequent analysis.

For the miRNA-perturbed gene expression profiles which comprise only one sample in any one class (‘transfection’ or ‘non-transfection’, ‘non-transfection’ also known as ‘control’), we applied ‘fold change’ to measure the extent of differential expression of each probe (fold change ≥ 2). The expression profiles which consist of greater than or equal to two samples in each class are conducted differential expression analysis using significance analysis of microarray (SAM, FDR ≤ 0.05). We built a response signature that contains differentially expressed probes and a ranked response pattern which is a full list of probes ranked from the most up-regulated to the most down-regulated by fold change or SAM statistics.

### Small molecule-perturbed gene expression profiles

cmap (build 02) contains 6100 genome-wide gene expression profiles that comprise the transcriptional responses to 1309 small molecules in cultured human cell lines. To compare the treatment group and control group, all the gene expression measurements were converted to amplitudes. Amplitude (*a*) is used to measure the extent of the differential expression of a given probe.


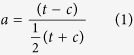


Where *t* is the expression value for the treatment and *c* is the expression value for the control. If *a* > 2/3, the expression is increased on treatment, i.e. *t* > 2*c*; if *a* < −2/3, the expression is decreased on treatment, i.e. *t* < 0.5*c*.

Analogous to miRNA-perturbed gene expression profile, we also assigned a response signature and a ranked response pattern to each small molecule. It’s worth noting that there are different gene expression profiles for the same small molecule due to various experiment conditions (such as cell lines, concentrations and batches). We considered several gene expression profiles for the same small molecule as independent datasets (also referred as “instances”), producing individual response signature and ranked response pattern.

In total, the small molecule-related response signature, as well as small molecule-related ranked response pattern is created for each small molecule-perturbed gene expression profile. The miRNA-related response signature and the miRNA-related ranked response pattern are constructed for each miRNA-perturbed gene expression profile.

### The Enrichment Score (ES) of small molecule/miRNA-related response signature relative to miRNA/small molecule-related ranked response pattern

The Enrichment Score (ES) is the maximum deviation from zero of *P*_*hit*_ – *P*_*miss*_.


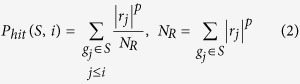



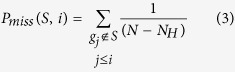


Here, *S* is the aforementioned response signature, which is the probes related to the phenotypic distinction (small molecule perturbation or miRNA perturbation), and represents the transcriptional responses to small molecule (or miRNA). 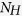 is the number of genes in the gene set. *L* is the ranked response pattern, which signifies the genome-wide transcriptional profile. *L* comprises *N* probe members. For the *i*_th_ member in *L*, we evaluate the fraction of probes in *S* (“hit”) weighted by their correlation and the fraction of probes not in S (“miss”) which are both present up to the given position *i* in *L*. We set parameter *p* = 1, weighting the probes in *S* by their correlation with perturbation (small molecule or miRNA) normalized by the sum of the correlations over all of the probes in *S*^25^.

For both small molecule and miRNA, we divided the response signature into up-regulated probe sets and down-regulated probe sets to calculated *ES*, respectively. The up-regulated or down-regulated probes of the miRNA-related response signature were searched within the ranked response pattern of small molecule, resulting in two enrichment scores (ES^up^_mir2sm_, ES^down^_mir2sm_), respectively. Analogously, up-regulated and down-regulated probes of small molecule-related response signature were also searched within the ranked response pattern of miRNA, producing two enrichment scores (ES^up^_sm2mir_, ES^down^_sm2mir_), respectively. We implemented the computational procedure of enrichment score with R program.

### The similarity of small molecule-perturbed and miRNA-perturbed gene expression profiles (total enrichment score)

For a small molecule *i* and miRNA *j*, we firstly obtained ES^up^_sm2mir_, ES^down^_sm2mir_, ES^up^_mir2mir_, ES^down^_mir2mir_ as above stated. Then, we combined ES^up^_sm2mir_ and ES^down^_sm2mir_ to Total Enrichment Score (TES_sm2mir_), and combined ES^up^_mir2sm_, ES^down^_mir2sm_ to Total Enrichment Score (TES_mir2sm,_). Lastly, we used the mean of TES_sm2mir_ and TES_mir2sm_ to measure the TES_(*i*,*j*)_ of the pair of small molecule *i* and miRNA *j*.


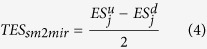



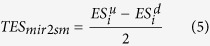



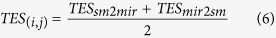


Here, *ES*_*x*_^*y*^(with x ∈ {*i*,*j*}, y ∈ {*u*_*s*_,*d*_*s*_} for small molecule and y ∈ {*u*_*m*_,*d*_*m*_} for miRNA) ranges in [−1, 1]. *ES*_*x*_^*y*^ is the enrichment score of the *y* (up or down) response signature with respect to the ranked response pattern of *x*. It quantifies how much a set of genes (or probes) is at the top of a ranked full list. The closer this measure is to 1, the closer the genes are to the top of the ranked full list. The closer the value to −1, the closer the genes are to the bottom of the ranked full list.

TES_(*i*,*j*)_ also ranges in [−1, 1]. It comprehensively quantifies how much the genes (or probes) in the up-regulated or down-regulated gene sets of (*u* or *d*) relative to small molecule *i* are placed at the top or bottom of the miRNA *j* and how much the genes (or probes) in the up-regulated or down-regulated gene sets (*u* or *d*) relative to miRNA *j* are placed at the top or bottom of the small molecule *i.* The closer these two statements are to the truth, the farther to 0 is the value of TES_(*i*,*j*)_, that is to say the larger is the absolute value of TES_(*i*,*j*)_.

### Pairwise association score (AS) of small molecules and miRNAs

Since multiple gene expression profiles are relative to one small molecule (aforementioned ‘instances’), the final Association Score (AS) for a pair of small molecule and miRNA is a composite score by combining TES of all the instances corresponding to the same small molecule with one miRNA via Kolmogorov-Smirnov (KS) test:


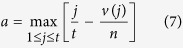



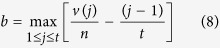



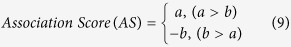


Here, *t* is the number of instances corresponding to one small molecule. *n* is the number of all the instances for all small molecules (*n* = 6100). *V*(*j*) is the *j*th element of the vector *V*, and denotes the position of the *j*th instance which are in the set of *t* instances in the ordered list of all *n* instances (*j* = 1,2,…,*t*). All the *n* instances are ranked in decreasing order according to the magnitude of TES.

Additionally, we performed a permutation test for each pair of small molecule and miRNA. We calculated 1000 random ASs generated by randomly sampling probes in ranked response pattern to form response signature while keeping the number of the response signature per small molecule or miRNA unchanged. *p*-value is the fraction of the absolute value of the random AS which is larger than that of the observed AS.

## Additional Information

**How to cite this article**: Meng, F. *et al.* Psmir: a database of potential associations between small molecules and miRNAs. *Sci. Rep.* **6**, 19264; doi: 10.1038/srep19264 (2016).

## Supplementary Material

Supplementary files
