# Supplementary material for: Psmir: a database of potential associations between small molecules and miRNAs
Source: Sci Rep. 2016 Jan 13;6:19264. doi: 10.1038/srep19264 (PMC4713048; doi:10.1038/srep19264)
Supplement: Supplementary files [file srep19264-s1.doc]

**Psmir: a database of potential associations between small molecules and miRNAs**

Fanlin Meng^1,†^, Jing Wang^1,†^, Feng Yang^1^, Xiaowen Chen^1^, Shuyuan Wang^1^, Enyu Dai^1^, Xuexin Yu^1^, Dianming Liu^1^, and Wei Jiang^1,*^

^1^ College of Bioinformatics Science and Technology, Harbin Medical University, Harbin 150081, P. R. China

^†^ Equal contribution

*To whom correspondence should be addressed.

**Table S1. Details of miRNA-perturbed data. The table provided the detail information for data of** **miRNA perturbation. Column No represents the serial number of data. Column miRNA represents the miRNA of perturbation. Column GSE is the GEO series number. Column** **Condition is the condition of miRNA perturbed. Column Mimics/Inhibitor is one miRNA mimic (or inhibitor)** **which was transfected into cells or cell lines. Column Cells/Cell lines represented which cell/cell lines was processed in one set of data.** **Column** **Platform is the platform that one set of data used, what’s more, these platforms were all Affymetrix HG-133-generation platforms.**

| No | miRNA | GSE | Condition | Mimics/  Inhibitor | Cells/Cell lines | Platform |
| --- | --- | --- | --- | --- | --- | --- |
| 1 | miR-221/222 | GSE10890 | 72h | M | MCF-7 | GPL570 |
| 2 | miR-155 | GSE13296 |  | I | Dendritic Cells (DC) | GPL570 |
| 3 | miR-7 | GSE14537 |  | M | HEK293 | GPL570 |
| 4 | miR-9 | GSE15749 |  | M | MCF-7 | GPL570 |
| 5 | miR-9* | GSE15749 |  | M | MCF-7 | GPL570 |
| 6 | miR-34a | GSE16674 |  | M | K562 | GPL570 |
| 7 | miR-210 | GSE16962 |  | M | HUVEC | GPL570 |
| 8 | miR-210 | GSE16962 |  | I | HUVEC | GPL570 |
| 9 | miR-145 | GSE18625 |  | M | DLD-1 cell line | GPL570 |
| 10 | miR-125b-2 | GSE19680 |  | M | CD34+-HSPCs | GPL570 |
| 11 | miR-34a | GSE21832 |  | M | MDA-MB-231 | GPL570 |
| 12 | miR-155 | GSE22002 | 32h | M | HeLa | GPL570 |
| 13 | miR-1 | GSE22002 | 32h | M | HeLa | GPL570 |
| 14 | miR-155 | GSE22002 | 12h | M | HeLa | GPL570 |
| 15 | miR-1 | GSE22002 | 12h | M | HeLa | GPL570 |
| 16 | miR-99a | GSE26332 |  | M | C4-2 | GPL570 |
| 17 | miR-7 | GSE27431 |  | M | HEY | GPL570 |
| 18 | miR-128 | GSE27431 |  | M | HEY | GPL570 |
| 19 | miR-31 | GSE28810 |  | M | U251 | GPL570 |
| 20 | miR-101 | GSE31397 |  | M | MCF-7 | GPL570 |
| 21 | miR-124 | GSE32876 |  | M | PDGFRA amplified neurospheres | GPL570 |
| 22 | miR-132 | GSE32876 |  | M | PDGFRA amplified neurospheres | GPL570 |
| 23 | miR-448 | GSE32876 |  | M | PDGFRA amplified neurospheres | GPL570 |
| 24 | miR-433 | GSE32876 |  | M | PDGFRA amplified neurospheres | GPL570 |
| 25 | miR-380 | GSE32876 |  | M | PDGFRA amplified neurospheres | GPL570 |
| 26 | miR-143 | GSE33420 |  | M | DLD-1 | GPL570 |
| 27 | miR-376a | GSE34454 |  | M | SW1783 | GPL570 |
| 28 | miR-376a | GSE34455 |  | M | U87 | GPL570 |
| 29 | miR-124 | GSE6207 | 4h | M | HepG2 | GPL570 |
| 30 | miR-124 | GSE6207 | 16h | M | HepG2 | GPL570 |
| 31 | miR-124 | GSE6207 | 24h | M | HepG2 | GPL570 |
| 32 | miR-124 | GSE6207 | 32h | M | HepG2 | GPL570 |
| 33 | miR-124 | GSE6207 | 72h | M | HepG2 | GPL570 |
| 34 | miR-124 | GSE6207 | 120h | M | HepG2 | GPL570 |
| 35 | miR-155 | GSE9264 |  | M | 293 cells | GPL570 |
| 36 | miR-26b | GSE12091 |  | M | HeLa | GPL571 |
| 37 | miR-98 | GSE12092 |  | M | HeLa | GPL571 |
| 38 | miR-206 | GSE31620 |  | M | LNCaP | GPL571 |
| 39 | miR-106b | GSE34893 |  | M | LNCaP | GPL571 |
